# Measuring Spanish Comprehension in Infants from Mixed Hispanic Communities Using the IDHC: A Preliminary Study on 16-Month-Olds

**DOI:** 10.3390/bs8120117

**Published:** 2018-12-15

**Authors:** Sandy L. Gonzalez, Eliza L. Nelson

**Affiliations:** Department of Psychology, Florida International University, Miami, FL 33199, USA; elnelson@fiu.edu

**Keywords:** bilingualism, infants, language, development

## Abstract

The MacArthur Inventario del Desarrollo de Habilidades Comunicativas: Primeras Palabras y Gestos (IDHC) is a widely-used parent report measure for infant Spanish language comprehension. The IDHC was originally created for use with infants of Mexican background. According to the U.S. 2017 census, however, about 37% of U.S. Hispanics are not of Mexican origin. In Miami-Dade, a large county in South Florida, 98% of Hispanics do not identify Mexico as their country of origin. IDHC use in mixed Hispanic communities such as Miami may be problematic due to differences in dialect and object labels. This study explored whether excluding IDHC words flagged as unknown or not commonly used by adults from mixed Hispanic communities affects bilingual infants’ vocabulary size. Data were collected from Hispanic 16-month-old infants (*N* = 27; females = 13) from a mixture of Latin American backgrounds residing in Miami, FL, USA, and compared to archival data from the IDHC Mexican norming sample (*N* = 60; females = 31). Findings indicate significant differences in the rate of comprehension between the two samples with infants from mixed Latin American backgrounds demonstrating lower rates of comprehension for words flagged as unknown/uncommon. Moreover, Spanish vocabulary scores for infants from mixed Hispanic communities were significantly lower compared to the Mexican norming sample. Use of total vocabulary score (i.e., Spanish + English) attenuated these issues in administrating the IDHC to bilingual infants from mixed Hispanic communities. Results suggest that comprehension of some IDHC words is influenced by Hispanic family background. These preliminary findings highlight potential issues in IDHC administration that require further investigation in additional samples spanning the full age range of the IDHC and from a range of socioeconomic backgrounds to effectively tune how we assess infant Spanish language comprehension to cultural differences.

## 1. Introduction

As of 2016, one-quarter of all children residing in the United States identify as Hispanic [1]. The Hispanic population’s growth largely stems from births, indicating that the number of Hispanic children in the U.S. will continue to grow regardless of changes in immigration patterns [2]. The shift in U.S. demographics necessitates a greater focus on Hispanic children’s early language development. Examining early language ability is imperative because prior research has found that gaps in language development may appear as young as two years of age [3]. Language comprehension, in particular receptive vocabulary, can be indexed early in development prior to language production [4,5]. Delays or difficulties in word comprehension have been tied to emergent literacy skills [6,7,8,9]. Improved understanding of language comprehension in Hispanic infants would provide insight into one of the earliest links in their language development trajectories.

Most research on language development in Hispanic infants has taken a pan-ethnic approach, with little discussion on potential differences between countries that speak Spanish as a primary language. The MacArthur Inventario del Desarrollo de Habilidades Comunicativas: Primeras Palabras y Gestos (IDHC; [10,11]) is a widely-used infant Spanish language comprehension measure for children 8–18 months of age [12,13,14]. Parents are given a checklist of 428 vocabulary words and asked to indicate whether their child comprehends words on the list. The IDHC is a more reliable measure of comprehension compared to other parent-report tools (e.g., diaries, interviews; [5,11]) because the IDHC relies on recognition rather than retrieval. The IDHC has high internal consistency (Cronbach’s alpha = 0.94) and high test-retest reliability (*r* = 0.97 for vocabulary comprehension) [10], as well as convergent validity with measures of vocabulary production [15]. The IDHC was created and normed using a Mexican sample from Southern California and Mexico [10,11]. However, about 37% of U.S. Hispanics do not identify Mexico as their country of origin [16]. This percentage is increased in some major metropolitan areas. New York, New Jersey, Washington, DC/Maryland/Virginia, Orlando, and Miami are in the top 60 metropolitan areas in the U.S. with a predominantly non-Mexican Hispanic population [17].

Hispanics from different Latin American countries of origin may label similar objects differently [18]. For example, the word “straw” in English may be labeled as “pitillo”, “pajita”, “paja”, “popote”, “sorbet”, “canita”, or “absorbent” in Spanish. Hispanics from different countries of origin may also value learning some concepts earlier than others, which could lead to differences in the timing of comprehension of certain words across nationalities. These differences could impact the child’s IDHC score. Research comparing the MacArthur–Bates Communicative Development Inventories (CDI) and the Australian English adaptation of the CDI (i.e., OZI) found that using the CDI in an Australian sample resulted in lower language scores compared to using the OZI [19]. Critically, evidence indicates that Hispanic subgroups can differ across development, particularly in the case of language [20,21,22]. Thus, use of assessments tuned to cultural differences is important when measuring language development. Adaptations of the IDHC for use with Cuban [14], Chilean [23], and Colombian [24] children have been developed. However, the creation of separate measures per nationality does not address the nature of the problem in the U.S., where a Hispanic community sample may include participants from a range of countries of origin. Although some research on bilingual language development has used Hispanic samples with a mixture of parental Latin American nationalities, Hispanic diversity has not been the primary research question (e.g., [13,25]).

What is well known from the literature on language development in bilingual populations is that it is critically important to measure language development across all the languages to which a child is regularly exposed [13,14,26,27,28]. In many cases, measuring only one language will result in an incomplete picture of bilinguals’ overall language development [12,25]. When measuring bilingual language development, two analytical techniques are common: (1) calculating total vocabulary score, and (2) correlating language exposure and vocabulary size. Use of a single vocabulary (e.g., English only) to compare vocabulary size in bilingual samples to monolingual samples results in a “gap”, where bilinguals tend to have a smaller vocabulary size in that language compared to monolinguals [26]. Using total vocabulary scores, researchers can ameliorate the gap by generating a combined vocabulary score using separate measures per language. Typically, researchers combine the CDI and IDHC to obtain total vocabulary scores in samples of English-Spanish bilingual infants [13,14]. Additionally, the amount of exposure in one language is positively correlated with vocabulary size in that specific language [25,29,30,31]. Most studies have used vocabulary scores per language for correlation with the percentage of language exposure [13,25,31,32]. Recently, DeAnda and colleagues [12] used relative vocabulary scores where the raw vocabulary score for the child’s dominant language was divided by the child’s total vocabulary score, yielding a percentage of how much that child’s dominant vocabulary is part of his/her total vocabulary. Relative vocabulary score was significantly correlated with child percentage of dominant language exposure. Taken together, understanding language comprehension requires accurate measurement of infants’ vocabulary.

It is unclear whether the words themselves on the IDHC may affect parent reporting on language comprehension in English-Spanish bilingual infants in families of non-Mexican origin. The aim of the present study was to compare infant comprehension of words on the IDHC between archival data from the Mexican norming sample and a sample of U.S. Hispanic infants from families with varied countries of origin. As a preliminary study, we explored response rates to words on the IDHC from parents of 16-month-old Hispanic infants in Miami, Florida, a city in the U.S. known for its diverse and heterogeneous Hispanic community, where 98% of Hispanics are of non-Mexican origin [33]. Given colloquial knowledge of differences in word use by Spanish speakers, we hypothesized that there would be a difference in reported language comprehension between the archival Mexican sample and the mixed Miami sample. We predicted that the mixed Miami sample would be characterized by significantly less comprehension for IDHC words that adult English-Spanish bilinguals identified as unknown or not commonly used as compared to response rates in the archival Mexican sample. We then explored whether excluding unknown/uncommon words affected vocabulary scores or altered the expected relation between vocabulary size and language exposure. Results are discussed as preliminary evidence justifying a larger coordinated effort in the field by bilingual infant researchers to renorm the IDHC for use in heterogeneous Hispanic communities.

## 2. Materials and Methods

### 2.1. Participants

Two samples of 16-month-old infants were compared for the purpose of this study: archival data of monolingual Spanish infants from Mexican background who completed the IDHC (*N* = 60, 31 female) and a newly-collected mixed Hispanic sample that completed the IDHC and the CDI (*N* = 27, 13 female). Data for the Mexican sample were obtained through Wordbank, an open-source database with archived data from the various language versions of the MacArthur–Bates Communicative Development Inventories [10,34]. Participating families in the mixed Hispanic sample were recruited from Miami-Dade county from a list of eligible families who had participated in unrelated previous studies with other lab groups on campus; flyers left at daycares, pediatrician offices, and indoor child recreation places; through postcard mailings; and postings on social media pages/groups focused on parenting.

All families in the mixed Hispanic sample identified their infant as Hispanic/Latino, and at least one parent identified as Hispanic/Latino. Eighty-four percent of infants were white, and 16% were of mixed race. One additional parent did not report race. Yearly family income ranged from less than $15,000–$100,000 or more, with $50,000–$74,999 as the median income level. Maternal education level ranged from high school or GED to graduate degree, with Bachelor’s degree or equivalent 4-year undergraduate degree as the median level of education. Paternal education level ranged from high school or GED to graduate degree, with Associate’s degree or equivalent 2-year undergraduate degree as the median level of education.

As part of the demographics questionnaire, parents in the mixed Hispanic sample were asked about their child’s country of origin, their own country of birth, and their familial country of origin (if parents were born in the U.S. to parents who immigrated to the U.S.) to gauge the diversity of family backgrounds in our sample. All of the infants were U.S. born. Table 1 displays the maternal and paternal countries of birth, and Table 2 displays maternal and paternal familial countries of origin. Although the majority of mothers and fathers were Cuban born/of Cuban background, there was still a broad range of Latin American nationalities represented in the sample. About 56% of parent dyads were of mixed familial countries of origin (e.g., a mother of Venezuelan background, a father of Cuban background). About 52% of mothers were immigrants to the U.S.; 33.3% were first-generation immigrants (i.e., their parents immigrated to the U.S., but they themselves were born in the U.S.), and 14.8% were second generation or older. About 60% of fathers immigrated to the U.S.; 37% were first-generation immigrants, and 3.7% were second generation or older. The mixed Hispanic sample reflects the diversity in countries of origin and immigrant generations seen in the Miami Hispanic/Latino community [18,33,35].

### 2.2. Procedures for the Mixed Hispanic Sample

Families in the mixed Hispanic sample portion of the study completed a phone interview and two parent language checklists online. A caregiver gave informed consent for their family to participate in the study. The Florida International University Institutional Review Board approved the following procedures (IRB-14-0348). Families were compensated with a $10 gift card for their time.

#### 2.2.1. Phone Interview

Parents in the mixed Hispanic sample completed an initial phone interview on or near the date of their child turning 16 months (±15 days, mean age at phone interview = 15.74 months, *SE* = 0.09, range = 15 months, 27 days to 16 months, 15 days). Demographic information was collected during the phone interview. Language exposure data using the Language Exposure Assessment Tool (LEAT) were also collected. The LEAT allows for quantification of language exposure through a structured parent interview regarding their child’s typical exposure to languages from each caregiver over time. The LEAT was completed on an Excel sheet equipped with pre-determined and protected equations permitting automatic generation of language exposure data [30]. Based on parent input, the LEAT Excel sheet calculated the child’s exposure to each language in their environment. The phone interview lasted approximately 30 min.

#### 2.2.2. Online Measures

Following completion of the phone interview, parents were then emailed a link to complete the Spanish language checklist (i.e., IDHC) and the English language checklist (i.e., CDI) online via Qualtrics. These measures are copyrighted. Links to the publisher can be found in Table A1. Parents had one week from the date of the phone interview to complete the online forms. In an effort to follow reported guidelines for adaptation of CDI measures, parents completed the IDHC and were allowed to provide structured feedback of the words on the checklist [36]. Words on the IDHC remained the same as the original version. However, beyond the usual choices of “comprende” (i.e., understands) and “comprende y dice” (i.e., understands and says), parents were also given the choice to respond to words with “todavia no comprende” (i.e., doesn’t understand this word yet), “comprende pero con otro nombre” (i.e., understands but with another name), and “yo no comprendo esta palabra” (i.e., I don’t understand this word). The option of “doesn’t understand this word yet” is different from the original paper form of the IDHC where parents do not respond to words their child does not know. Having the parent confirm that their child did not understand a word was chosen because the online format could have resulted in missed/inaccurate clicking. When parents responded to an item with the option “understands with another name”, blank fields were generated and parents could provide the alternative word their child used for the given word on the checklist. The option of “I don’t understand this word” was added to identify words that the parent themselves did not know on the IDHC and were unable to identify with a different Spanish label. This option permitted for identification of unknown/uncommon words based on parent feedback. At the end of each section of the IDHC, parents could also provide any words that did not appear in that section that their child knew. The IDHC was always given first in the online portion of the study to ensure parent feedback on IDHC words was optimized given the length of completing both checklists. After the IDHC, parents were asked to complete the CDI. Possible responses on the CDI matched those found on the paper form (i.e., “understands” and “understands and says”) and also included an option of “my child doesn’t understand this word yet” to verify clicks in the online format. The online portion of the study took approximately 1 h.

### 2.3. IDHC Word Analysis

A comprehensive list was generated of words that were identified by parents on the IDHC as “(child) understands with another name” or “I don’t understand this word”. In addition, 10 adult Spanish-English bilinguals living in Miami representing a range of Latin American countries of origin independently reviewed the IDHC checklist and identified any unknown words or known words for which they used an alternate label based on their family background. Conservatively, we selected only the words that overlapped between these two expert sources for further analysis.

To facilitate comparisons between the archival Mexican norming sample and our newly collected mixed Miami sample, we recoded parent responses in the mixed Hispanic group into the original IDHC responses. Words parents identified as “understands but with another name” or “I (parent) don’t understand this word” were recoded as “doesn’t understand” in line with our research question to examine whether certain words may affect IDHC administration and scoring.

### 2.4. Statistics

Fisher’s exact tests were conducted to examine differences in rate of comprehension between the archival Mexican norming sample and the mixed Hispanic sample for each of the unknown/uncommon IDHC words. Westfall and Young’s method [37] for resampling-based multiple testing using bootstrapping was applied to reduce false discovery rate or type I error. Separate independent samples *t*-tests were also used to compare the mixed Hispanic sample and the archival Mexican norming sample on: (1) Spanish-only vocabulary size using all of the IDHC words; (2) total vocabulary score for the bilingual mixed Hispanic sample (i.e., Spanish using all IDHC words + English); (3) updated Spanish-only vocabulary scores that excluded identified unknown/uncommon IDHC words with significantly different rates of reported comprehension; and (4) updated total vocabulary score (i.e., also excluding identified unknown/uncommon IDHC words) for the bilingual mixed Hispanic sample. All reported independent sample *t*-test results utilized Levene’s test for the equality of variances to ensure that that results were less prone to type 1 error due to unequal sample sizes between groups [38]. Pearson’s correlations were used to assess the relation between vocabulary size (with and without the list of unknown/uncommon IDHC words) and language exposure. The significance level was set at 0.05 for all analyses. Analyses were conducted in SAS 9.4 [39] and SPSS 23 [40].

## 3. Results

All parents completed the LEAT and IDHC (*N* = 27). Due to attrition, a subset of parents (*N* = 17) also completed the CDI. Spanish exposure for infants in the mixed Hispanic sample ranged from 15–100% (*M* = 60.47% ± 5.48), and English exposure ranged from 0–86% (*M* = 39.63% ± 5.50) as calculated by the LEAT. Of the 428 words on the IDHC checklist, 40 were mutually identified as unknown or not commonly used in the mixed Hispanic sample by parents and other adult Spanish-English bilinguals (Table A2). Of the 40 words, 16 were found to have significantly different rates of reported comprehension between the two samples (Table 3). Overall, the mixed Hispanic sample demonstrated lower rates of comprehension on the words that were identified as unknown/uncommon as compared to the archival Mexican sample.

**Figure 1:**
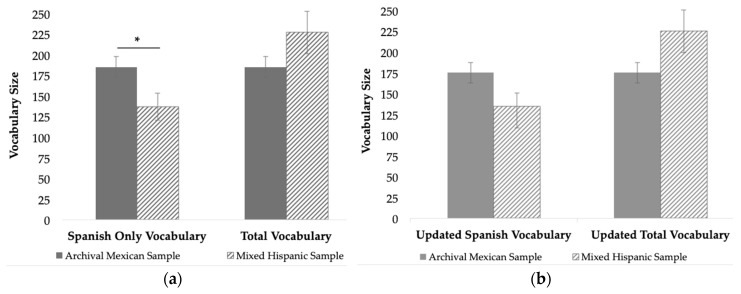
Mean Spanish-only vocabulary size and total vocabulary size for the monolingual archival Mexican sample and the bilingual mixed Hispanic sample (**a**) using the standard IDHC and (**b**) subtracting unknown/uncommon IDHC words. * *p* < 0.05. See the text for details.

An independent samples *t*-test comparing Spanish only vocabulary size using all of the IDHC words identified a significant difference between the monolingual archival Mexican sample and the bilingual mixed Hispanic sample, *t*(80) = −2.059, *p* = 0.043, *d* = 0.54 (Figure 1a). The bilingual mixed Hispanic sample had a smaller Spanish vocabulary compared to the archival monolingual Mexican sample. Comparing the two samples using total vocabulary score for the bilingual mixed Hispanic sample (i.e., Spanish + English), there was no significant difference in vocabulary size between the two groups, *t*(75) = 1.528, *p* = 0.131, *d* = 0.40 (Figure 1a).

To measure the potential effect of the unknown/uncommon IDHC words on vocabulary score, we generated updated Spanish-only vocabulary scores that excluded the 16 unknown/uncommon IDHC words with significantly different rates of reported comprehension between the two samples. An independent samples *t*-test on updated Spanish-only vocabulary size did not find the standard significant difference between the monolingual archival Mexican sample and the bilingual mixed Hispanic sample that was typically reported in the literature, *t*(80) = −1.806, *p* = 0.075, *d* = 0.54 (Figure 1b). We again compared the two samples using the updated total vocabulary score for the bilingual mixed Hispanic sample and found no significant difference in vocabulary size between the groups, *t*(75) = 1.871, *p* = 0.065, *d* = 0.50 (Figure 1b). There were no differences in variance based on Levene’s test for the equality of variances for all of the reported independent samples *t*-tests, indicating homoscedasticity and reduced type 1 error between samples of different sizes [38].

The relationship between vocabulary size and language exposure (as reported on the LEAT) within the mixed Hispanic bilingual sample was further analyzed with and without the list of 16 unknown/uncommon IDHC words. Using the standard IDHC, there was no significant correlation between raw Spanish vocabulary and Spanish exposure (*r* = 0.296, *p* > 0.05) or English exposure (*r* = −0.300, *p* > 0.05). Relative vocabulary size was also calculated (dominant vocabulary/total vocabulary, where dominant vocabulary is vocabulary in the language receiving >50% exposure). There was a significant positive correlation between relative vocabulary size and dominant language exposure, *r* = 0.604, *p* = 0.01. We then reexamined the relation between vocabulary size and language exposure using the updated Spanish vocabulary score without the unknown/uncommon IDHC words. There was no significant correlation between the updated Spanish vocabulary score and Spanish (*r* = 0.293, *p* > 0.05) or English exposure (*r* = −0.296, *p* > 0.05). We replicated the correlation between relative vocabulary and dominant language exposure using the updated Spanish score, *r* = 0.612, *p* < 0.009.

## 4. Discussion

The aim of the current study was to test the feasibility of administering the Mexican-normed IDHC [10,11], a Spanish language parent checklist designed to measure infant comprehension, in a mixed Hispanic community sample with participants from a range of Latin American countries of origin. We hypothesized that family background impacts Spanish language use, which in turn could impact accurate measurement. Using two sources of Spanish-English bilingual expertise, we generated a list of IDHC words that were reported to be either unknown or not commonly used. We then tested for differences in the rate of comprehension on these words between the archival Mexican norming sample and our mixed Hispanic sample. As predicted, parents in the mixed Hispanic sample reported significantly lower comprehension for a subset of the IDHC words. Including the IDHC words where comprehension differed significantly between the two samples in calculating Spanish-only vocabulary size replicated a previously-described ‘gap’ between the monolingual Mexican norming sample and the bilingual mixed Hispanic sample (e.g., [13]). The mixed Hispanic sample had a significantly smaller vocabulary size compared to the monolingual Spanish sample. Including English vocabulary for bilinguals by calculating total vocabulary size ameliorated this gap as expected, providing further confirmation for measuring all languages to which a child is exposed [13,14,26,27,28]. Our study adds to the literature [13,14] by showing that excluding the subset of IDHC words that were flagged as unknown/uncommon resulted in no difference in vocabulary size between the two groups of infants. Thus, removal of just 16 words from the IDHC out of 428 total words closed the gap between monolingual and bilingual Spanish vocabulary size.

Overall, most words on the IDHC checklist were not found to be problematic for Hispanic bilinguals from mixed family backgrounds, despite the fact that the measure was not originally normed with a heterogeneous sample of Spanish-speaking parents. However, these results highlight that the current version of the IDHC includes some words that researchers who work with diverse Hispanic populations should be cautious of, as they may prove to be problematic for parents to report on. Both parents and other adult Spanish-English bilinguals who participated in this study reported that they were unfamiliar with multiple words on the IDHC and identified that they used different labels for other words. This finding supports the need for multiple “experts” (i.e., parents and other individuals with experience in the local dialect) for input for situations where investigators may opt for in-house adaptations of the IDHC until updated and more inclusive norms are available.

Importantly, not all of the words on the list generated by experts had significantly different comprehension rates between the archival Mexican norming sample and the mixed Hispanic sample. In other words, comprehension rates for some of the items that were flagged as potentially problematic were within the same range regardless of family background. For example, a word like “borrego” was understood at a low rate in both groups, suggesting that the differences between the two samples on specific words were a product of dialect differences, and not of bilingual exposure. Moreover, our bilingual experts independently generated many more potentially problematic words than we analyzed. Due to the large number of words on the IDHC, we chose a more conservative strategy to focus on words that overlapped between experts. In a larger sample, additional words could and should be examined. We are also not aware of any recent studies that have surveyed parents of monolingual Spanish infants on the suitability of the IDHC content.

The unknown/uncommon words represent <4% of the total IDHC, yet subtracting these words from the Spanish vocabulary scores made a difference in the vocabulary size and eliminated the gap between monolingual and bilingual infants. One caveat for using the IDHC with bilinguals from mixed nationalities is that researchers must be aware that the gap seen in Spanish-only comparisons may be partially due to IDHC content. For our bilingual sample, calculating total vocabulary (i.e., combining Spanish and English vocabulary knowledge) compensated for any disadvantages in IDHC administration from the lack of culturally neutral items. However, if researchers using the IDHC are interested in Spanish language metrics specifically, they may want to consider comparing vocabulary size with and without the list of unknown/uncommon words or undertake their own analyses of word use by their local sample against archival data, which is freely available.

While removing the unknown/uncommon IDHC words had an effect on comparisons of vocabulary size between groups, there was no effect of including/excluding these words on the analyses examining the links between vocabulary size and Spanish exposure. There is surprisingly limited information in the literature on how parent-reported levels of Spanish exposure relate to raw vocabulary scores. According to Pearson and colleagues [31], parent-reported Spanish exposure is correlated with Spanish vocabulary size, even when children have lower rates of exposure, such as 10%. Hoff and colleagues [13,41] discussed English, but not Spanish exposure, in relation to raw vocabulary size per language. More recent work by this group found that maternal education level in Spanish was significantly related to children’s Spanish skill (but not English skill), while maternal education level in English was significantly related to children’s English skill (but not Spanish skill) [42]. It is possible that the quality of caregiver input, rather than the quantity, can provide additional insight into the complexities of English-Spanish bilingual development (e.g., [25]).

Our results on relative vocabulary and dominant vocabulary exposure support prior work [12]. Infants’ amount of dominant language exposure was positively correlated with their relative vocabulary size in their dominant language, with and without the unknown/uncommon IDHC words. Each child’s experience and word knowledge is relative because individual bilinguals experience different amounts of exposure [43]. By creating a proportion of dominant vocabulary size, relative vocabulary score helps control for total vocabulary size, while still allowing researchers to describe how vocabulary size relates to exposure. Relative vocabulary scores allow for more equal comparison across bilinguals by accounting for individual variation in vocabulary knowledge, which could explain why removing certain words from the calculation had no effect. 

The demographics of the families in our sample may have influenced our results. It is clear from a large body of work that maternal education and family SES matters for child language development and future academic outcomes [44,45,46,47,48]. For example, research by Hammer and colleagues analyzing the relation between vocabulary and parental demographic factors in a sample of Head Start participating children found that maternal education was a significant predictor of child vocabulary comprehension during Head Start [45]. Median maternal education within our sample was a Bachelor’s degree or equivalent four-year undergraduate degree, indicating that our mixed Hispanic sample was highly educated. Moreover, our mixed Hispanic sample had a wide range of family income, but median income was moderately high (e.g., $50,000–$74,999). It is possible that the higher SES found in our sample affected reported child vocabulary. Moreover, recent work by Hoff and colleagues highlighted that the language in which the highest level of maternal education is obtained matters for language outcomes per language in bilingual children [42]. It is important to note however that the higher SES observed in our sample is not uncommon in samples of Hispanic children residing in South Florida, given the high diversity of immigrant populations from a range of Latin American countries and their diverse reasons for immigration [18,42].

It is important to emphasize the preliminary nature of the current study. In particular, the sample size for the diverse Hispanic sample was not large, which may have affected our results. However, a sample size of 27 falls within the sample size range suggested by the MacArthur Bates Advisory Board when gathering initial feedback from parents on a CDI measure for adaptation, in line with what we have presented here [36]. Moreover, sample sizes between the bilingual mixed Hispanic sample and the Mexican norming sample were different, but no differences in variance based on Levene’s test for the equality of variances were detected, indicating homoscedasticity. If unequal variances were found and not corrected, the type 1 error rate would have been affected [38]. Moreover, the effect sizes for analyses comparing the two samples were within the moderate range, indicating that the magnitude of the differences between the two groups was still large enough to detect given the small sample size [49].

Overall, we want to stress that the results presented here are only an initial step towards tuning the IDHC to cultural differences. Further research is needed to disentangle the relation between language outcomes as measured by the IDHC and participant cultural background. A larger coordinated effort in the field from bilingual infant researchers is needed to renorm the IDHC for use in heterogeneous Hispanic communities. Future work should aim to examine the IDHC across its range of use from 8–18 months, as well as a range of socioeconomic backgrounds, and in larger sample sizes. It is imperative that researchers heed this call for IDHC improvement, as measurement of Spanish language development is becoming increasingly relevant due to growth in the population of Hispanic children in the U.S. [1,2].

## 5. Conclusions

Overall, our results suggest that parent-reported comprehension of some IDHC words is influenced by Hispanic family background. We recommend using total vocabulary score to attenuate this issue in bilingual samples with mixed backgrounds. We caution that our sample size was small and limited to one time point. Ultimately, a measure that can be used across infants and toddlers of different Latin American backgrounds would advance our understanding of both Spanish monolingual language development, as well as English-Spanish bilingual development in Hispanic children.

## Figures and Tables

**Table 1:** Maternal and paternal country of birth in mixed Hispanic sample. ^1^

Maternal Country of Birth	Paternal Country of Birth
USA (48.1%)	USA (37%)
Cuba (14.8%)	Cuba (29.6%)
Dominican Republic (7.4%)	Nicaragua (11.1%)
Venezuela (7.4%)	Puerto Rico (7.4%)
Chile (3.7%)	Argentina (3.7%)
Colombia (3.7%)	Chile (3.7%)
Mexico (3.7%)	Venezuela (3.7%)
Peru (3.7%)	Haiti (3.7%)
Puerto Rico (3.7%)	
Lithuania (3.7%)	

^1^ For the mother born in Lithuania, she reported moving to Cuba as an infant, had Cuban parents, was a fluent Spanish speaker, and never learned Lithuanian. For the father born in Haiti, the mother reported that he only spoke English to their infant and did not report exposure to any language besides English and Spanish.

**Table 2:** Maternal and paternal familial country of origin in mixed Hispanic sample.

Maternal Familial Country of Origin	Paternal Familial Country of Origin
Cuba (22.2%)	Cuba (44.4%)
USA (14.8%)	Nicaragua (11.1%)
Colombia (7.4%)	Puerto Rico (7.4%
Dominican Republic (7.4%)	Argentina (3.7%)
Mexico (7.4%)	Chile (3.7%)
Puerto Rico (7.4%)	Haiti (3.7%)
Chile (3.7%)	Jamaica (3.7%)
El Salvador (3.7%)	Mexico (3.7%)
Peru (3.7%)	Peru (3.7%)
Venezuela (3.7%)	Venezuela (3.7%)

**Table 3:** Reported comprehension differs between samples for a subset of the MacArthur Inventario del Desarrollo de Habilidades Comunicativas: Primeras Palabras y Gestos (IDHC) words.

IDHC Word	Mixed Sample Understands	Mexican Sample Understands	X^2^ Statistic	*p*-Value
1. Guaguá	25.93%	91.67%	39.30	<0.001
2. Carreola	7.69%	56.76%	17.89	<0.001
3. Chile	3.85%	41.67%	12.30	0.005
4. Paleta	15.38%	58.33%	13.50	0.007
5. Calcetines	29.63%	76.67%	17.50	<0.001
6. Calzón	3.85%	41.67%	12.30	0.005
7. Pañal	51.85%	86.76%	12.33	0.018
8. Playera	3.85%	40.00%	11.50	0.011
9. Suéter	15.38%	55.00%	11.61	0.016
10. Botella/Mamila	30.77%	86.67%	26.87	<0.001
11. Estufa	7.69%	51.67%	14.83	<0.001
12. Lavabo	0.00%	43.33%	16.15	<0.001
13. Tina	19.23%	58.33%	11.15	0.020
14. Tortillitas	11.54%	60.00%	17.19	<0.001
15. Aventar	23.08%	70.00%	16.20	0.002
16. Fuchi	7.69%	73.33%	31.42	<0.001

## Appendix A

**Table A1:** Links to parent language checklist measures.

IDHC: https://products.brookespublishing.com/MacArthur-Inventario-Del-Desarrollo-de-Habilidades-Comunicativas-Inventario-P49.aspx
CDI: https://products.brookespublishing.com/MacArthur-Bates-Communicative-Development-Inventories-CDI-Words-and-GesturesDesktop-Scannable-English-P82.aspx

IDHC: Inventario del Desarrollo de Habilidades Comunicativas: Primeras Palabras y Gestos. CDI: MacArthur-Bates Communicative Development Inventories: Words and Gestures.

**Table A2:** Unknown/uncommon IDHC words identified by the mixed Hispanic sample.

IDHC Word	Section of the IDHC	Word Category
1. ¡Am!	Sonidos de Cosas y Animales	Sound
2. ¡Ay!	Sonidos de Cosas y Animales	Sound
3. Guaguá ^1^	Sonidos de Cosas y Animales	Sound
4. Tutú	Sonidos de Cosas y Animales	Sound
5. Borrego	Animales (de Verdad y de Juguete)	Noun
6. Guajalote	Animales (de Verdad y de Juguete)	Noun
7. Puerco	Animales (de Verdad y de Juguete)	Noun
8. Carreola ^1^	Vehículos (de Verdad y de Juguete)	Noun
9. Atole	Alimentos y Bebidas	Noun
10. Chile ^1^	Alimentos y Bebidas	Noun
11. Jugo	Alimentos y Bebidas	Noun
12. Paleta ^1^	Alimentos y Bebidas	Noun
13. Aretes	Ropa	Noun
14. Calcetines ^1^	Ropa	Noun
15. Calzón ^1^	Ropa	Noun
16. Lentes	Ropa	Noun
17. Pañal ^1^	Ropa	Noun
18. Playera ^1^	Ropa	Noun
19. Shorts	Ropa	Noun
20. Suéter ^1^	Ropa	Noun
21. Chichi/Pecho	Partes del Cuerpo	Noun
22. Botella/Mamila ^1^	Utensilios de la Casa	Noun
23. Cobija	Utensilios de la Casa	Noun
24. Chupón/Chupete	Utensilios de la Casa	Noun
25. Bacinica	Muebles y Cuartos	Noun
26. Cajón	Muebles y Cuartos	Noun
27. Estufa ^1^	Muebles y Cuartos	Noun
28. Lavabo ^1^	Muebles y Cuartos	Noun
29. Recámara	Muebles y Cuartos	Noun
30. Refrigerador	Muebles y Cuartos	Noun
31. Tina^1^	Muebles y Cuartos	Noun
32. Resbaladilla	Lugares y Objetos Fuera de la Casa	Noun
33. Acerrín	Rutina Diaria, Reglas Sociales y Juegos	Verb
34. Hacer la meme	Rutina Diaria, Reglas Sociales y Juegos	Verb
35. Ojitos	Rutina Diaria, Reglas Sociales y Juegos	Verb
36. Tengo Manita	Rutina Diaria, Reglas Sociales y Juegos	Verb
37. Tortillitas ^1^	Rutina Diaria, Reglas Sociales y Juegos	Verb
38. Aventar ^1^	Acciones y Procesos (Verbos)	Verb
39.Bonita	Cualidades y Atributos	Adjective
40. Fuchi ^1^	Cualidades y Atributos	Adjective

^1^ Denotes words with significantly-different comprehension rates. See the text for details.
